# Oral Application of Mother's Own Milk for Reducing Necrotizing Enterocolitis in Preterm Infants: An Updated Meta-Analysis of RCTs

**DOI:** 10.1155/2023/7378064

**Published:** 2023-04-07

**Authors:** Bo Peng, Lei Yu, Jing Qian, Baoying Zheng, Yi Zhang, Chunmei Zhu

**Affiliations:** ^1^Department of Respiratory, Children's Hospital Affiliated to Capital Institute of Pediatrics, Beijing, China; ^2^Infection Management Division, Children's Hospital Affiliated to Capital Institute of Pediatrics, Beijing, China

## Abstract

**Background:**

Necrotizing enterocolitis (NEC) and late-onset sepsis (LOS) are the major contributors to mortality and morbidity in preterm infants. This updated meta-analysis was aimed to assess the effects of mother's milk on the incidence of NEC, LOS, and other clinical outcomes in preterm infants.

**Methods:**

PubMed, Embase, and the Cochrane library were searched for papers published up to October 2022.

**Results:**

A total of 13 RCTs with 1330 infants were included in the final analysis. Significant difference in NEC (stage 2 or 3) was found between the intervention group and the control group (RR = 0.508, 95% CI: 0.314–0.822, and *P*=0.008). The incidence of proven LOS (RR = 0.809, 95% CI: 0.610–1.071, and *P*=0.139) and death (RR = 0.800, 95% CI: 0.571–1.122, and *P*=0.196) was comparable between the two groups. Statistical differences in the incidence of proven or probable LOS (RR = 0.705, 95% CI: 0.577–0.862, and *P*=0.001) and length of hospitalization (WMD = −4.868, 95% CI: −6.608 to −3.128, and *P* < 0.001) between the intervention group and the control group were observed.

**Conclusions:**

The results of this updated meta-analysis showed that compared to the placebo, mother's milk provides better effects in reducing the incidences of NEC, proven or probable LOS, and the length of stay, whereas no significant benefit of mother's milk was observed in reducing the incidence of proven LOS and death.

## 1. Introduction

Necrotizing enterocolitis (NEC) and late-onset sepsis (LOS) are the major contributors to mortality and morbidity in premature infants [1]. According to previous research studies, the incidence of NEC is 2–7% among infants with gestational age (GA) <32 weeks and 5–22% among infants with birth weight (BW) <1000 g and the incidence of LOS among hospitalized infants varies geographically from 0.61 to 14.2% [2, 3]. Considerable evidence suggested that both NEC and LOS remained the major contributors to mortality rates as high as 20–30% and 13–19%, respectively, among preterm infants in neonatal intensive care units (NICUs) [4–6].

Mother's milk is the best first immune stimulator in infants, featuring the perfect species-specific nutrition, because it contains many types of protective agents and enhances neurodevelopmental outcomes [7, 8]. Colostrum is produced in the first few days postpartum and is rich in immune factors dynamically switching in accordance with the mothers' condition [9]. As the immune factors in human milk can protect infants from infection and provide them with antimicrobial, anti-inflammatory, and immunomodulatory functions, human milk is recognized as the most beneficial form of nutrition for infants [8, 10]. Numerous studies have focused on the value of colostrum in terms of preventing, improving, and curing diseases [11–13]. It has been shown that colostrum appears to lower the risk of LOS, feeding intolerance, and other complications of preterm labor [10, 14].

With advancements in neonatal care, the mortality in preterm infants has improved significantly. However, this has been associated with an increase in LOS and NEC. Although many meta-analyses have studied the effect of colostrum therapy on the incidence of NEC and LOS, previous evidence remains uncertain regarding the protective effect of colostrum on infants due to the paucity of sample size [15–17]. In addition, a previous meta-analysis also included studies that intervened with bovine milk or a commercial milk formula, which might cause potential bias in terms of the solitary effect of mother's milk. According to recent published studies, feeding with own mother's milk is shown to be protective against LOS and NEC in preterm infants [18]. And to date, several new studies investigating the effects of mother's milk on reducing the incidence of NEC and LOS have been published. However, the results are inconsistent [16, 19–21]. It is therefore necessary to increase the study population in order to draw more reliable conclusions. For these reasons, this updated meta-analysis was aimed to assess the effects of mother's milk on the incidence of NEC, LOS, and other clinical outcomes in preterm infants.

## 2. Materials and Methods

### 2.1. Ethical Statement

We developed the framework of the current systematic review and meta-analysis according to the recommendations issued by the Cochrane Collaboration for the purpose of ensuring the methodological quality because we did not register a formal protocol [22]. We did not impose ethical approval and patients' informed consent because all essential data in the current systematic review and meta-analysis were extracted from published studies.

### 2.2. Literature Search

This meta-analysis was conducted according to the Preferred Reporting Items for Systematic Reviews and Meta-Analyses (PRISMA) guidelines (Supplementary Material 1) [23]. Relevant clinical trials were searched based on the PICO process [24]. Randomized controlled studies published up to October 2022 were searched for in PubMed, Embase, and the Cochrane library using MeSH terms “Enterocolitis Necrotizing,” “Neonatal Sepsis,” “Infant,” “Colostrum,” and relevant key words (Supplementary Material 2). Relevant articles were searched and followed by screening based on the eligibility criteria: (1) population: infants; (2) interventions: breastfeeding with own mother's milk or placebo; (3) outcomes: the number of infants diagnosed with NEC and proven or probable LOS; (4) study type: randomized controlled studies published in scientific peer-reviewed journals; and (5) language: limited to English. No ethical consent was required because this study was performed based on previous data.

### 2.3. Data Extraction

Two investigators independently extracted the following items using the predesigned data extraction sheet: study characteristics (authors, year of publication, the country where the study performed, and sample size), patients' characteristics (dose of intervention, birth weight, gestation age, number of cases, and gender percent of infants), and outcomes (the primary outcome was the incidence of NEC, and the secondary outcomes were the incidence of proven or probable LOS, death, and the length of hospitalization). Any inconsistencies in data extraction were solved based on the consensus principle.

## 3. Outcome

The primary outcome was the incidence of NEC (diagnosed as Grade [25] II or higher according to well-validated classification criteria). The secondary outcomes were the incidence of death, LOS, all stage of NEC, and the length of stay. LOS, including proven and probable sepsis, was defined as sepsis occurring at >72 h of life.

### 3.1. Quality of the Evidence

The risk of bias of all included studies was assessed independently by two authors using the RoB-2 criteria [26]. The level of evidence was assessed by GRADE. Discrepancies in the assessment were resolved through discussion until a consensus was reached. The heterogeneity across the included studies was calculated using the *I*^2^ and *Q* statistics. The fixed-effect model was applied to investigate the effect of oral application of mother's own milk on the incidence of necrotizing enterocolitis, late-onset sepsis, and death, as we realized the RCTs included in our meta-analysis showed rather small heterogeneity in terms of the population (preterm infants with low birth weight) and the application of intervention (mostly received 0.2 ml of their own mothers' milk for 2 to 5 days). We also performed the sensitivity analysis by the random-effect model. For studies that did not present their results as means ± standard deviations, the results were estimated based on the reported parameters (median, standard error, IQR, or 95% CI) [27]. Potential publication bias was assessed by funnel plots and Egger's test if there were ≥10 studies included in the analysis of an outcome; otherwise, the funnel plots and Egger's test could yield misleading results and were not recommended [22].

## 4. Results

### 4.1. Selection and Characteristics of the Studies

**Figure 1:**
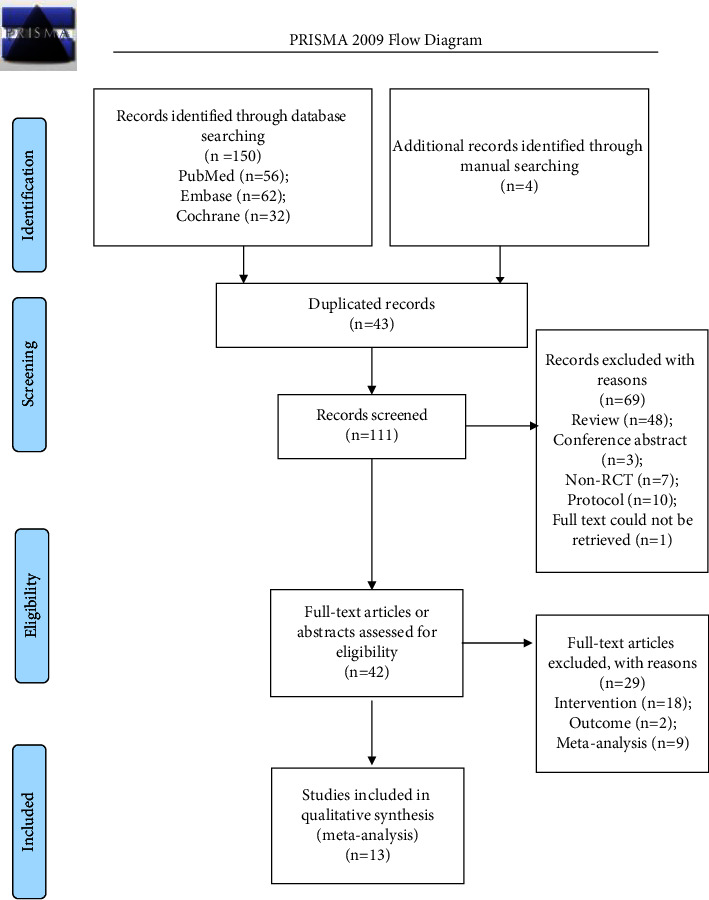
Study selection process.

Figure 1 presents the study inclusion process. A total of 154 trials were first retrieved, and 111 trials were left after removing duplicates. Then, 69 studies were excluded because of the type of article and unavailability of full-text articles. From the 42 studies left, after reviewing full-text articles, 29 were excluded (9 meta-analyses, 2 for outcomes, and 18 for the intervention). Therefore, a total of 13 RCTs [18, 20, 21, 28–37] were included in the final analysis (Table 1). At the end of our research, a total of 1330 infants were included in our analyses, with approximately 660 infants in each group.

### 4.2. Quality of the Included Studies

The assessment for the risk of bias for the included studies is presented in Table 2. Among the 13 randomized controlled trials [18, 20, 21, 28–37], the overall risk of bias was low in 8 studies [20, 21, 28–31, 33–36]. Two studies that did not describe the process of randomization and blinding were degraded according to the ROB-2 criteria [16, 32]. All 13 studies were graded as a low risk of bias regarding the terms of missing outcome data and the measurement of outcomes. Some concerns were raised in the assessment for bias arising from the randomization process in four studies [18, 28, 31, 36] and bias due to deviations from intended interventions in six studies [16, 21, 31–33, 36]. Overall, we identified one study that suffered from a high risk of bias [32], which was further analyzed in sensitivity analysis. The assessment for the level of evidence using GRADE is presented in Supplementary Material 3.

### 4.3. Effect of Intervention on the Incidence of NEC

**Figure 2:**
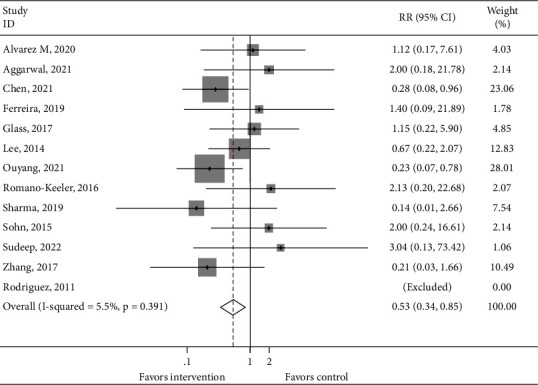
Forest plot comparing the incidence of necrotizing enterocolitis between the intervention group and the control group.

A total of 12 studies reported the incidence of NEC. One study [28] did not clarify the stage of NEC in the analysis which was excluded from the primary outcome. Therefore, only 11 studies were left for the incidence of NEC (stage 2 or 3). A significant difference was found between the intervention group and the control group (RR = 0.508, 95% CI: 0.314–0.822, 1208 infants from 11 studies, and *P*_heterogeneity_=0.391, Figure 2).

### 4.4. Secondary Outcomes

**Figure 3:**
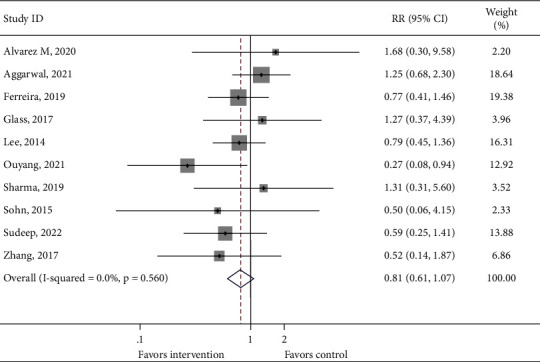
Forest plot comparing the incidence of proven late-onset sepsis between the intervention group and the control group.

**Figure 4:**
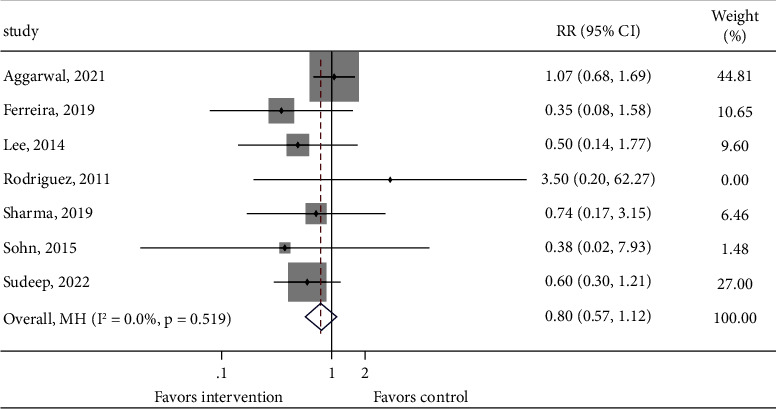
Forest plot comparing the incidence of death between the intervention group and the control group.

**Figure 5:**
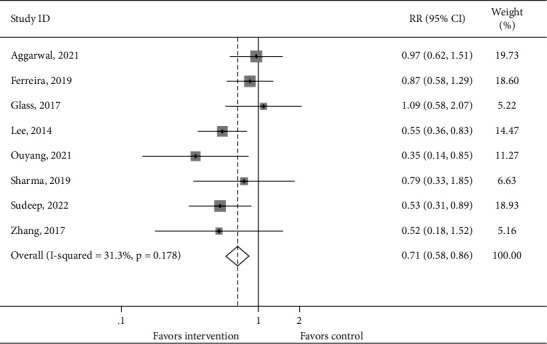
Forest plot comparing the incidence of proven or probable late-onset sepsis between the intervention group and the control group.

The incidence of proven LOS and death in both the treatment and control groups was reported in 10 and 9 cohorts of patients, respectively. No significant difference was found between the intervention group and the control group (Figures 3 and 4, proven LOS: RR = 0.809, 95% CI: 0.610–1.071, 1103 infants from 10 studies, and *P*_heterogeneity_=0.560; death: RR = 0.800, 95% CI: 0.571–1.122, 977 infants from 9 studies, and *P*_heterogeneity_=0.519). However, 8 cohorts of patients reported a statistically difference in the incidence of proven or probable LOS between the intervention group and the control group (RR = 0.705, 95% CI: 0.577–0.862, 1004 infants from 8 studies, and *P*_heterogeneity_=0.178, Figure 5). In addition, a significant difference was observed in the length of hospitalization between the intervention group and the control group (WMD = −4.868, 95% CI: −6.608 to −3.128, 1031 infants from 8 studies, and *P*_heterogeneity_=0.005, Supplementary Material 4).

### 4.5. Sensitivity Analysis and Publication Bias

The results of sensitivity analysis conducted by omitting one study at a time were similar in the combined results of NEC (stage 2 or 3), proven LOS, proven or probable LOS, and death, without great fluctuation, suggesting that pooled RRs were relatively stable (Supplementary Materials 5a–5d). Although we observed marginal significance in the analysis of NEC (stage 2 or 3), the direction of the combined results under the random-effect model was not significantly changed (Supplementary Materials 6a–6d). However, in the analysis of the length of stay, the combined result might be influenced by one single study (Supplementary Materials 5e and 6e). Publication bias for NEC (stage 2 or 3) and proven LOS were assessed by Egger's test and funnel plots (Supplementary Materials 7a and 7b).

## 5. Discussion

The clinical evidence regarding the strengths of feeding with mother's milk in infants has great implications for neonatal infection-related diseases. The results in this updated meta-analysis demonstrated that, compared to the placebo, mother's milk provides better effects in reducing the incidence of NEC, proven or probable LOS, and the length of stay among infants, whereas no significant difference was found in terms of the incidence of proven LOS and death between the two groups, indicating that despite the positive effect, additional treatments and substantial monitoring should be performed in clinical care.

The results of our analysis are partially congruent with those of the previous study. In a previous meta-analysis, Ma et al. [16] concluded that, compared to the placebo, oropharyngeal colostrum was associated with potential significance of NEC (OR = 0.51, 95% CI: 0.26–0.99, and *P*=0.05), a trend toward downregulating mortality (OR = 0.60, 95% CI: 0.34–1.08, and *P*=0.09), and proven sepsis (OR = 0.64, 95% CI: 0.40–1.01, and *P*=0.06). Despite our study yielding a similar result, it is worth noting that we included 5 newly published studies with over 700 more cases in our study. Moreover, we explicitly include studies comparing mother's milk in the intervention group and sterile water in the control group in order to avoid potential bias due to deviations from intended interventions. Another recent published meta-analysis [15] investigating the role of lactoferrin supplementation (using bovine lactoferrin or recombinant human lactoferrin in the intervention group) in preterm infants has suggested that lactoferrin supplementation without probiotics could significantly decrease the incidence of all LOS (proven or probable LOS, RR = 0.43; 95% CI: 0.29–0.62; *I*^2^ = 0%). But compared to the control group, the incidence of NEC (RR = 0.62, 95% CI: 0.29–1.33, and *I*^2^ = 43%) and all-cause mortality (RR = 0.74; 95% CI: 0.36–1.53, and *I*^2^ = 53%) was not significantly reduced in the intervention group. In 2020, Tao et al. [17] reported that oropharyngeal administration of colostrum does not reduce the incidence of NEC, LOS, and death in preterm infants, whereas they found that the duration of hospital stay (MD = −10.38, 95% CI = −18.47–2.29, *I*^2^ = 94.7%, and *P*=0.010) was significantly shortened in the intervention group. These results contradicted the results in our analysis. The possible explanation for this difference might also be their relatively small sample size and substantial heterogeneity across their studies.

In addition, it is important to note the feeding type, dose of intervention, and duration of breast milk feeding in this field. Some studies found that colostrum was effective in reducing the rate of NEC, revealing its effect in building up immunity [38–41]. Fortunately, the dose of breast milk was consistent throughout the included studies. But when attaching the importance to the feeding type, researchers also found that the oral cavities of some preterm infants are easily colonized by pathogens after being administered into the NICU and the use of oral rinses such as chlorhexidine, which might result in unpleasant adverse effects, or even death [42, 43]. In addition, preterm infants who have undeveloped digestive systems might be at greater risk of infection as they have to be breastfed via a nasogastric tube that bypasses their oropharynx. Thus, oral priming with colostrum is considered a better way to help preterm infants strengthen their immunity.

The results of this meta-analysis must be considered in light of its strength and limitations. First, to our knowledge, this updated meta-analysis included several newly published studies and approximately doubled the sample size of a previous meta-analysis, therefore providing more reliable results when comparing mother's milk and the placebo in reducing the incidence of severe clinical outcomes among infants. However, according to the quality assessment by the ROB-2 criteria, we found several studies that suffered from a high risk of bias regarding the randomization and blinding process. But the sensitivity analyses showed that the estimated parameters did not affect the conclusions. Second, as the dosage and duration of breast milk feeding might yield distinct heterogeneity between studies, the variation in the inclusion may lead to different clinical outcomes between studies. We decided to use the fixed-effect model throughout the entire analysis, as we realized that the RCTs included in our meta-analysis showed rather small heterogeneity in terms of the population (preterm infants with low birth weight) and intervention (mostly received 0.2 ml of their own mothers' milk for 2 to 5 days). Besides, based on the results of the *I*^2^ and *Q*-test for heterogeneity, we only found subtle-to-mild heterogeneity across studies. Third, after carefully reviewing the included studies, we found two studies that [21, 32] used donor mother's milk (DHM) during the intervention when mother's own milk (MoM) was not available for any reason. Fortunately, there was only a small fraction of patients using DHM as an alternative. Given the results of our analysis, we recommended that future studies should focus on the mechanism of potential immunological benefits of mother's milk and its effect on other neonatal diseases.

## 6. Conclusions

In conclusion, the results of this updated meta-analysis showed that, compared to the placebo, mother's milk provides better effects in reducing the incidences of NEC, proven or probable LOS, and the length of stay, whereas no significant benefit of mother's milk was observed in reducing the incidence of proven LOS and death.

## Figures and Tables

**Table 1:** Characteristics of the included studies.

Author, year	Country	BW, GA	Intervention	Intervention dose	Sample size		Male (%)	
					Intervention	Control	Intervention	Control
Alvarez, 2020	Spain	<1500 g, <32 weeks	MOM	0.2 ml/4 h/14 days	41	46	60	56.5
Aggarwal, 2021	India	<32 weeks	MOM and DHM	0.2 ml/3 h	128	128	53.1	45.4
Chen, 2021	China	<1000 g, <28 weeks	MOM	0.2 ml/4 h/5 days	51	53	52.94	47.17
Ferreira, 2019	Brazil	<1500 g, <34 weeks	MOM	0.2 ml/2 h/2 days	47	66	49	59
Glass, 2017	USA	<1500 g	MOM	0.2 ml/3 h/5 days	17	13	41	62
Lee, 2014	Korea	<28 weeks	MOM	0.2 ml/3 h/3 days	24	24	50	42
Ouyang, 2021	China	≤32 weeks	MOM	0.2 ml/3 h/10 days	127	125	51.2	62.4
Rodriguez, 2011	USA	<1000 g, <28 weeks	MOM	0.2 ml/2 h/2 days	9	6	77.8	50
Romano-Keeler, 2016	USA	<32 weeks	MOM and DHM	0.2 ml/6 h/5 days	48	51	50	41
Sharma, 2019	India	<1250 g, <30 weeks	MOM	0.2 ml/2 h/3 days	59	58	45.7	51.7
Sohn, 2015	USA	<1500 g, <30 weeks	MOM	0.2 ml/2 h/46 h	6	6	33.3	50
Sudeep, 2022	India	<31 weeks	MOM	0.2 ml/3 h	66	67	54.5	56.7
Zhang, 2017	China	<1500 g	MOM	0.2 ml/4 h/7 days	32	32	56.2	53.1

**Table 2:** Cochrane risk-of-bias assessment tool 2 (ROB-2) for randomized controlled trials.

Study	1	2	3	4	5	6
Alvarez, 2020	High	Some concerns	Low	Low	Low	Some concerns
Aggarwal, 2021	Low	Some concerns	Low	Low	Low	Low
Chen, 2021	Low	Low	Low	Low	Low	Low
Ferreira, 2019	Some concerns	Low	Low	Low	Low	Low
Glass, 2017	Some concerns	High	Low	Low	Low	Some concerns
Lee, 2014	Low	Low	Low	Low	Low	Low
Ouyang, 2021	Low	Low	Low	Low	Low	Low
Rodriguez, 2011	Some concerns	Some concerns	Low	Low	Low	Low
Romano-Keeler, 2016	High	Some concerns	Low	Low	High	High
Sharma, 2019	Low	Some concerns	Low	Low	Some concerns	Some concerns
Sohn, 2015	Some concerns	Some concerns	Low	Low	Some concerns	Some concerns
Sudeep, 2022	Low	Low	Low	Low	Low	Low
Zhang, 2017	Low	Low	Low	Low	Low	Low

(1) Bias arising from the randomization process; (2) bias due to deviations from intended interventions; (3) bias due to missing outcome data; (4) bias in measurement of the outcome; (5) bias in selection of the reported result; (6) overall bias.

## Data Availability

All data generated or analyzed during this study are included in this published article and its supplementary information files.

## Supplementary Materials

Supplementary Materials
*Supplementary Material 1.* PRISMA_2020_checklist. *Supplementary Material 2.* Literature search strategies in PubMed, Embase, and Cochrane library. *Supplementary Material 3.* Grading of Recommendations, Assessment, Development, and Evaluation (GRADE) criteria for study outcomes. *Supplementary Material 4.* Forest plot comparing the length of stay between the intervention group and the control group. *Supplementary Material 5.* Sensitivity analysis of (a) necrotizing enterocolitis, (b) proven late-onset sepsis, (c) proven or probable late-onset sepsis, (d) death, and (e) length of stay. *Supplementary Material 6.* Pooled results under the random-effect model (a) necrotizing enterocolitis, (b) proven late-onset sepsis, (c) proven or probable late-onset sepsis, (d) death, and (e) length of stay. *Supplementary Material 7.* Egger's test and funnel plots for (a) necrotizing enterocolitis and (b) proven late-onset sepsis.
