# Supplementary material for: Oral Application of Mother's Own Milk for Reducing Necrotizing Enterocolitis in Preterm Infants: An Updated Meta-Analysis of RCTs
Source: Evid Based Complement Alternat Med. 2023 Apr 7;2023:7378064. doi: 10.1155/2023/7378064 (PMC10104743; doi:10.1155/2023/7378064)
Supplement: Supplementary Materials — Supplementary Material 1. PRISMA_2020_checklist. Supplementary Material 2. Literature search strategies in PubMed, Embase, and Cochrane library. Supplementary Material 3. Grading of Recommendations, Assessment, Development, and Evaluation (GRADE) criteria for study outcomes. Supplementary Material 4. Forest plot comparing the length of stay between the intervention group and the control group. Supplementary Material 5. Sensitivity analysis of (a) necrotizing enterocolitis, (b) proven late-onset sepsis, (c) proven or probable late-onset sepsis, (d) death, and (e) length of stay. Supplementary Material 6. Pooled results under the random-effect model (a) necrotizing enterocolitis, (b) proven late-onset sepsis, (c) proven or probable late-onset sepsis, (d) death, and (e) length of stay. Supplementary Material 7. Egger's test and funnel plots for (a) necrotizing enterocolitis and (b) proven late-onset sepsis. [file 7378064.f1.zip › Supplementary material 2 (1).docx]

Additional File 2. Search strategy

Title: Oral application of mother’s own milk for reducing necrotizing enterocolitis in preterm infants: an updated meta-analysis of RCTs

**Date：2022/10**

| **PubMed** |  | **Search strategy** | **Numbers** |
| --- | --- | --- | --- |
| **Patient** | **#1** | "Infant"[Mesh] OR Infants OR "Infant, Newborn"[Mesh] OR Infants, Newborn OR Newborn Infant OR Newborn Infants OR Newborns OR Newborn OR Neonate OR Neonates | 1585090 |
| **Intervention** | **#2** | "Colostrum"[Mesh] OR Colostrums | 10186 |
|  | **#3** | "Lactoferrin"[Mesh] OR Lactotransferrin | 9709 |
|  | **#4** | #2 OR #3 | 19534 |
| **Outcome** | **#5** | "Enterocolitis, Necrotizing"[Mesh] OR Necrotizing Enterocolitis | 9938 |
|  | **#6** | "Neonatal Sepsis"[Mesh] OR Neonatal Sepses OR Sepses, Neonatal OR Sepsis, Neonatal OR Neonatal Late-Onset Sepsis OR Late-Onset Sepses, Neonatal OR Late-Onset Sepsis, Neonatal OR Neonatal Late Onset Sepsis OR Neonatal Late-Onset Sepses OR Sepses, Neonatal Late-Onset OR Sepsis, Neonatal Late-Onset | 12679 |
|  | **#7** | #5 OR #6 | 21200 |
| **Study** | **#8** | "Randomized Controlled Trials as Topic"[Mesh] OR Clinical Trials, Randomized OR Trials, Randomized Clinical OR Controlled Clinical Trials, Randomized | 731235 |
| **All** | **#9** | #1 AND #4 AND #7 AND #8 | 56 |

| Embase |  | Search strategy | Numbers |
| --- | --- | --- | --- |
| Patient | **#1** | infant OR infants OR 'newborn'/exp OR 'animals, newborn' OR 'child, newborn' OR 'full term infant' OR 'human neonate' OR 'human newborn' OR 'infant, newborn' OR 'neonatal animal' OR 'neonate' OR 'neonate animal' OR 'neonatus' OR 'newborn animal' OR 'newborn animals' OR 'newborn baby' OR 'newborn child' OR 'newborn infant' OR 'newly born animal' OR 'newly born baby' OR 'newly born child' OR 'newly born infant' | 1535906 |
| Intervention | **#2** | 'colostrum'/exp OR 'beestings (milk)' OR 'colostrium' OR 'foremilk' | 9451 |
|  | **#3** | 'lactoferrin'/exp OR 'iron lactoferrin' OR 'lactoferrine' OR 'lactotransferrin' OR 'liposomal lactoferrin' | 12172 |
|  | **#4** | #2 OR #3 | 21255 |
| Outcome | **#5** | 'necrotizing enterocolitis'/exp OR 'acute necrotising enterocolitis' OR 'acute necrotizing enterocolitis' OR 'enterocolitis necroticans' OR 'enterocolitis, acute necrotising' OR 'enterocolitis, acute necrotizing' OR 'enterocolitis, necrotising' OR 'enterocolitis, necrotizing' OR 'necrotising enterocolitis' | 14816 |
|  | **#6** | 'newborn sepsis'/exp OR 'neonatal sepsis' OR 'neonatal septicaemia' OR 'neonatal septicemia' OR 'newborn septicaemia' OR 'newborn septicemia' | 11895 |
|  | **#7** | #5 OR #6 | 25671 |
| Study | **#8** | 'randomized controlled trial (topic)'/exp OR 'pragmatic clinical trials as topic' OR 'randomized controlled trials' OR 'randomized controlled trials as topic' | 287635 |
| All | **#9** | #1 AND #4 AND #7 AND #8 | 62 |

| Cochrane |  | Search strategy | Numbers |
| --- | --- | --- | --- |
| Patient | **#1** | MeSH descriptor: [Infant] explode all trees | 35105 |
|  | **#2** | Infants | 37604 |
|  | **#3** | #1 OR #2 | 57983 |
|  | **#4** | MeSH descriptor: [Infant, Newborn] explode all trees | 17651 |
|  | **#5** | (Infants, Newborn) OR (Newborns) OR (Newborn) OR (Neonates) OR (Newborn Infants) OR (Newborn Infant) OR (Neonate) | 35477 |
|  | **#6** | #4 OR #5 | 35593 |
|  | **#7** | #3 OR #6 | 69129 |
| Intervention | **#8** | MeSH descriptor: [Colostrum] explode all trees | 133 |
|  | **#9** | Colostrums | 13 |
|  | **#10** | #8 OR #9 | 146 |
|  | **#11** | MeSH descriptor: [Lactoferrin] explode all trees | 291 |
|  | **#12** | Lactotransferrin | 8 |
|  | **#13** | #11 OR #12 | 298 |
|  | **#14** | #10 OR #13 | 439 |
| Outcome | **#15** | MeSH descriptor: [Enterocolitis, Necrotizing] explode all trees | 314 |
|  | **#16** | Necrotizing Enterocolitis | 2214 |
|  | **#17** | #15 OR #16 | 2214 |
|  | **#18** | MeSH descriptor: [Neonatal Sepsis] explode all trees | 86 |
|  | **#19** | (Neonatal Late-Onset Sepses) OR (Neonatal Late-Onset Sepsis) OR (Neonatal Late Onset Sepsis) OR (Late-Onset Sepses, Neonatal) OR (Sepsis, Neonatal Late-Onset) OR (Late-Onset Sepsis, Neonatal) OR (Sepses, Neonatal Late-Onset) OR (Sepsis, Neonatal Early-Onset) OR (Neonatal Early Onset Sepsis) OR (Sepses, Neonatal Early-Onset) OR (Neonatal Early-Onset Sepsis) OR (Early-Onset Sepsis, Neonatal) OR (Neonatal Early-Onset Sepses) OR (Early-Onset Sepses, Neonatal) OR (Neonatal Sepses) OR (Sepses, Neonatal) OR (Sepsis, Neonatal) | 2598 |
|  | **#20** | #18 OR #19 | 2598 |
|  | **#21** | #17 OR #20 | 3939 |
| Study | **#22** | MeSH descriptor: [Randomized Controlled Trials as Topic] explode all trees | 15210 |
|  | **#23** | (Controlled Clinical Trials, Randomized) OR (Clinical Trials, Randomized) OR (Trials, Randomized Clinical) | 1009167 |
|  | **#24** | #22 OR #23 | 1010052 |
| All | **#25** | #7 AND #14 AND #21 AND #24 | 35 (32 trials) |
