# Supplementary material for: Oral Application of Mother's Own Milk for Reducing Necrotizing Enterocolitis in Preterm Infants: An Updated Meta-Analysis of RCTs
Source: Evid Based Complement Alternat Med. 2023 Apr 7;2023:7378064. doi: 10.1155/2023/7378064 (PMC10104743; doi:10.1155/2023/7378064)
Supplement: Supplementary Materials — Supplementary Material 1. PRISMA_2020_checklist. Supplementary Material 2. Literature search strategies in PubMed, Embase, and Cochrane library. Supplementary Material 3. Grading of Recommendations, Assessment, Development, and Evaluation (GRADE) criteria for study outcomes. Supplementary Material 4. Forest plot comparing the length of stay between the intervention group and the control group. Supplementary Material 5. Sensitivity analysis of (a) necrotizing enterocolitis, (b) proven late-onset sepsis, (c) proven or probable late-onset sepsis, (d) death, and (e) length of stay. Supplementary Material 6. Pooled results under the random-effect model (a) necrotizing enterocolitis, (b) proven late-onset sepsis, (c) proven or probable late-onset sepsis, (d) death, and (e) length of stay. Supplementary Material 7. Egger's test and funnel plots for (a) necrotizing enterocolitis and (b) proven late-onset sepsis. [file 7378064.f1.zip › Supplementary material 3 (1).docx]

Additional File 3. Grading of Recommendations, Assessment, Development, and Evaluation (GRADE) criteria for study outcomes.

| **Certainty assessment** | | | | | | | **№ of patients** | | **Effect** | | **Certainty** | **Importance** |
| --- | --- | --- | --- | --- | --- | --- | --- | --- | --- | --- | --- | --- |
| **№ of studies** | **Study design** | **Risk of bias** | **Inconsistency** | **Indirectness** | **Imprecision** | **Other considerations** | **Intervention** | **Control** | **Relative (95% CI)** | **Absolute (95% CI)** |  |  |
| **The incidence of necrotizing enterocolitis (assessed with: incidence)** | | | | | | | | | | | | |
| 11 | randomised trials | serious^a^ | not serious | not serious | serious^b^ | none | 23/603 (3.7%) | 45/605 (6.9%) | **RR 0.54** (0.34 to 0.85) | **32 fewer per 1,000** (from 45 fewer to 10 fewer) | ⨁⨁◯◯ Low | CRITICAL |
| **The incidence of proven late-onset sepsis (assessed with: incidence)** | | | | | | | | | | | | |
| 10 | randomised trials | serious^a^ | not serious | not serious | serious^b^ | none | 68/542 (12.5%) | 89/561 (15.9%) | **RR 0.82** (0.63 to 1.06) | **29 fewer per 1,000** (from 59 fewer to 10 more) | ⨁⨁◯◯ Low | CRITICAL |
| **The incidence of death (assessed with: incidence)** | | | | | | | | | | | | |
| 7 | randomised trials | serious^a^ | not serious | not serious | serious^b^ | none | 50/483 (10.4%) | 64/494 (13.0%) | **RR 0.81** (0.59 to 1.11) | **25 fewer per 1,000** (from 53 fewer to 14 more) | ⨁⨁◯◯ Low | CRITICAL |
| **The incidence of proven or probable late-onset sepsis (assessed with: incidence)** | | | | | | | | | | | | |
| 8 | randomised trials | serious^a^ | not serious | not serious | serious^b^ | none | 105/495 (21.2%) | 157/509 (30.8%) | **RR 0.74** (0.62 to 0.88) | **80 fewer per 1,000** (from 117 fewer to 37 fewer) | ⨁⨁◯◯ Low | IMPORTANT |
| **The length of hospitalization (assessed with: days)** | | | | | | | | | | | | |
| 8 | randomised trials | serious^a^ | very serious^c,d^ | not serious | serious^b^ | none | 506 | 525 | - | **0**  (0 to 0 ) | ⨁◯◯◯ Very low | IMPORTANT |

**CI:** confidence interval; **RR:** risk ratio

#### Explanations

a. The components of placebos were different across studies.

b. Donor's milk and own mother's milk were both applied.

c. The I-squared for this outcome is 65.5%

d. Sensitivity analysis showed inconsistent results.
