# Supplementary material for: Oral Application of Mother's Own Milk for Reducing Necrotizing Enterocolitis in Preterm Infants: An Updated Meta-Analysis of RCTs
Source: Evid Based Complement Alternat Med. 2023 Apr 7;2023:7378064. doi: 10.1155/2023/7378064 (PMC10104743; doi:10.1155/2023/7378064)
Supplement: Supplementary Materials — Supplementary Material 1. PRISMA_2020_checklist. Supplementary Material 2. Literature search strategies in PubMed, Embase, and Cochrane library. Supplementary Material 3. Grading of Recommendations, Assessment, Development, and Evaluation (GRADE) criteria for study outcomes. Supplementary Material 4. Forest plot comparing the length of stay between the intervention group and the control group. Supplementary Material 5. Sensitivity analysis of (a) necrotizing enterocolitis, (b) proven late-onset sepsis, (c) proven or probable late-onset sepsis, (d) death, and (e) length of stay. Supplementary Material 6. Pooled results under the random-effect model (a) necrotizing enterocolitis, (b) proven late-onset sepsis, (c) proven or probable late-onset sepsis, (d) death, and (e) length of stay. Supplementary Material 7. Egger's test and funnel plots for (a) necrotizing enterocolitis and (b) proven late-onset sepsis. [file 7378064.f1.zip › Supplementary material 4.pdf]

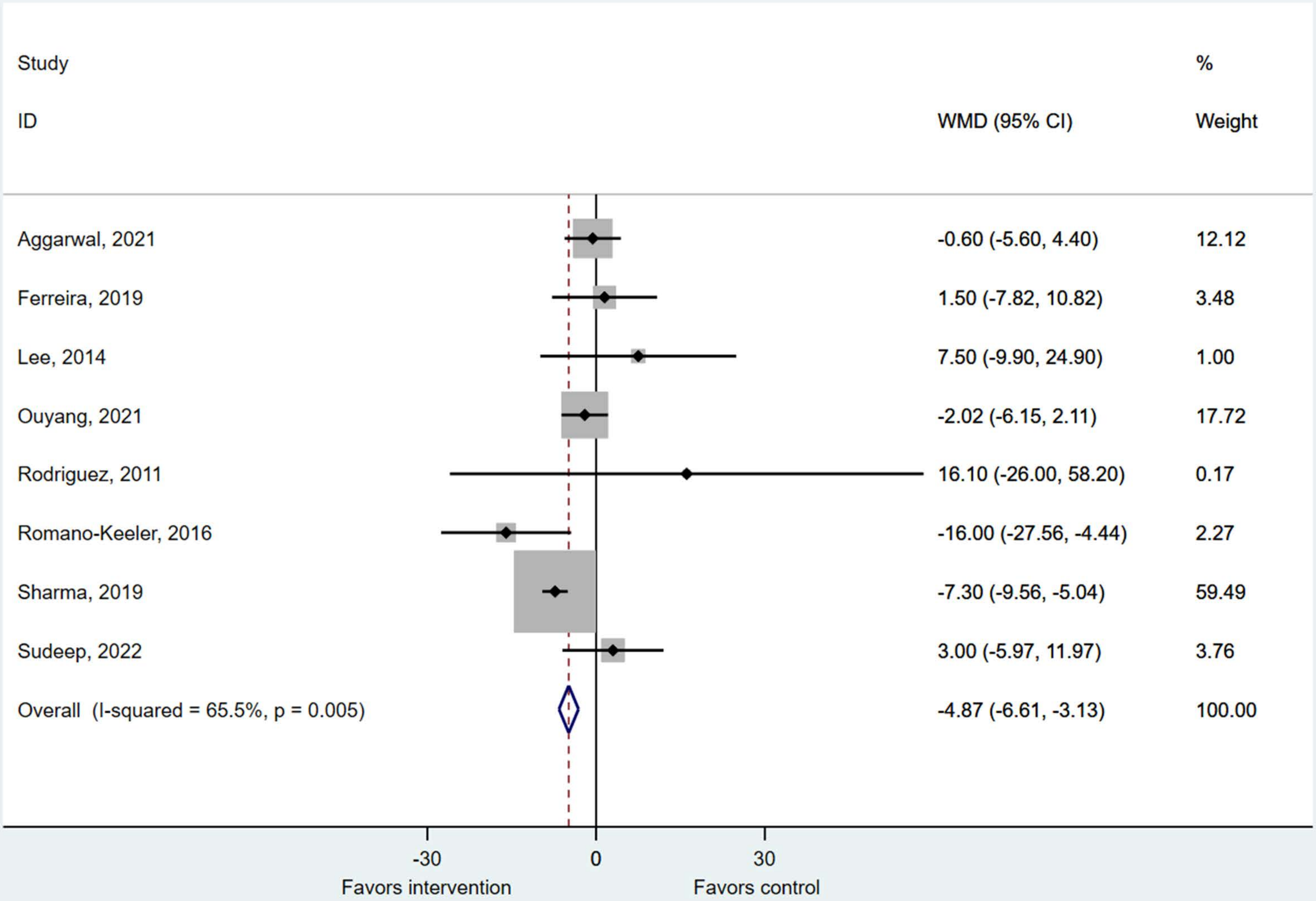

Study
ID
%
WMD (95% CI)
Weight
Aggarwal, 2021
-0.60 (-5.60, 4.40)
12.12
Ferreira, 2019
1.50 (-7.82, 10.82)
3.48
Lee, 2014
7.50 (-9.90, 24.90)
1.00
Ouyang, 2021
-2.02 (-6.15, 2.11)
17.72
Rodriguez, 2011
16.10 (-26.00, 58.20)
0.17
Romano-Keeler, 2016
-16.00 (-27.56, -4.44)
2.27
Sharma, 2019
-7.30 (-9.56, -5.04)
59.49
Sudeep, 2022
3.00 (-5.97, 11.97)
3.76
Overall (I-squared = 65.5%, p = 0.005)
-4.87 (-6.61, -3.13)
100.00
-30
0
30
Favors intervention
Favors control
